# c-erbB-2 expression in benign and malignant breast disease.

**DOI:** 10.1038/bjc.1988.239

**Published:** 1988-10

**Authors:** B. A. Gusterson, L. G. Machin, W. J. Gullick, N. M. Gibbs, T. J. Powles, C. Elliott, S. Ashley, P. Monaghan, S. Harrison

**Affiliations:** Institute of Cancer Research, Haddow Laboratories, Surrey, UK.

## Abstract

**Images:**


Br. J. Cancer (1988), 58, 453–457 © The Macmillan Press Ltd., 1988

### c-*erbB-2* expression in benign and malignant breast disease

B.A. Gusterson[1], L.G. Machin[2], W.J. Gullick[3], N.M. Gibbs[4], T.J. Powles[5], C. Elliott[5], S. Ashley[5], P. Monaghan[1] & S. Harrison[1]

[1]*Institute of Cancer Research, Haddow Laboratories, Clifton Avenue, Sutton, Surrey, SM2 5PX;* [2]*Department of Pathology, St. Helier Hospital, Wrythe Lane, Carshalton, Surrey;* [3]*Imperial Cancer Research Fund, Hammersmith Hospital, Du Cane Road, London W12;* [4]*Department of Pathology, Royal Surrey County Hospital, Guildford, Surrey; and* [5]*Royal Marsden Hospital, Downs Road, Sutton, Surrey, SM2 5PX, UK.*

**Summary** An antibody, 21N, raised against a synthetic peptide from the predicted sequence of the c-*erbB-2* protein has been used immunocytochemically in a retrospective study of formalin fixed paraffin embedded breast biopsies. Fourteen out of 103 infiltrating ductal carcinomas exhibited positive membrane staining. Fifty-four of these tumours had lymph node involvement of which nine contained stained cells. These were all cases where the primary tumour was positive. In this series there was no correlation between c-*erbB-2* overexpression and lymph node status. In five of the positive cases studied there was an associated *in situ* component which was also positively stained. Ten out of 24 pure intraduct carcinomas showed membrane staining, but none of the 149 benign conditions studied, which included 22 radial scars and 13 cases of atypical ductal proliferation, demonstrated the pattern of staining associated with overexpression. It is concluded that the c-*erbB-2* protein is overexpressed in a minority (~14%) of infiltrating ductal carcinomas and only in cells that are cytologically malignant. Overexpression of c-*erbB-2* is considered in relation to pathogenesis.

The c-*erbB-2* gene is a normal cellular gene which is present on chromosome 17 (Fukushige *et al.*, 1986) and encodes a protein which has close sequence homology with the epidermal growth factor receptor (Coussens *et al.*, 1985; Bargmann *et al.*, 1986*a*). This gene was originally identified in ethyl nitrosourea induced rat neuroblastoma cell lines as an activated oncogene called *neu* (Padhy *et al.*, 1982; Bargmann *et al.*, 1986*b*). *Neu* was shown to encode a 185,000 molecular weight protein, which from its structure (Coussens *et al.*, 1985; Bargmann *et al.*, 1986*a,b*) and localisation to the membrane (Drebin *et al.*, 1984) suggests that it may be a receptor for an as yet unidentified growth factor. In rat cell lines the oncogenic-*neu* gene differs from the cellular-*neu* gene by a single base change (glutamic acid to valine) in the putative transmembrane region (Bargmann *et al.*, 1986*b*).

Clinical interest in the gene in breast cancer was initiated by a report from Slamon's group which indicated that amplification of this gene may be related to poor prognosis, with the claim that amplification alone had greater prognostic value than hormonal-receptor status in lymph node-positive patients (Slamon *et al.*, 1987). Work on cell lines had previously shown that in human breast carcinoma the c-*erbB-2* gene could be overexpressed by a number of mechanisms, one of which was amplification (Kraus *et al.*, 1987). More recently of perhaps more biological significance was the observation by Di Fiore *et al.* (1987) that c-*erbB-2* needed to be overexpressed in NIH/3T3 cells (5–10 times) to be transforming. The fact that the cellular gene could be transforming without the point mutation supported the view that overexpression of this gene in breast carcinomas could have significance for the pathogenesis of this disease. Subsequent to the Slamon paper other groups, including our own, have confirmed the high incidence of c-*erbB-2* amplification in breast cancer (van de Vijver *et al.*, 1987; Venter *et al.*, 1987; Varley *et al.*, 1987; Zhou *et al.*, 1987) and two of these have also indicated a correlation between amplification and poor prognosis (Varley *et al.*, 1987) and between gene amplification and lymph node metastases (Zhou *et al.*, 1987).

On the basis of the c-*erbB-2* protein sequence, Gullick's group produced two polyclonal antibodies to c-terminal peptides (Gullick *et al.*, 1987). Using one of the antibodies it was demonstrated that amplification, which was present in 12 out of 36 tumours, correlated with overexpression of the c-*erbB-2* protein, measured immunocytochemically and by Western blotting (Venter *et al.*, 1987). In a subsequent publication using the same series of tumours we have shown that strong membrane staining using one of the antibodies, 21N, correlated with gene amplification (Gusterson *et al.*, 1987). Measurement of EGF receptor and oestrogen receptor status in this same series showed no significant correlation with c-*erbB-2* overexpression (Gusterson *et al.*, 1987). This preliminary study, which was carried out on formalin fixed paraffin embedded material, indicated the utility of antibody 21N for retrospective analyses of tumours and benign breast diseases. The present paper describes an extensive immunocytochemical study of infiltrating ductal carcinomas, lymph node metastases, intraduct carcinomas and a range of benign breast disease, including 22 radial scars and 13 cases of atypical ductal proliferation.

### Materials and methods

### *Tissues used*

This study was carried out on 103 cases of infiltrating ductal carcinoma, selected into two groups on the basis of the lymph node status of the patients at the time of primary breast surgery and division of the patients into those that had relapsed within one year and those that were disease free after five years.

In addition 24 cases of intraduct carcinoma were examined and 150 examples of benign breast disease taken from material obtained at the Guildford Breast Screening Programme. Access to this material gave us the opportunity of examining 22 stellate scars and 13 cases of atypical ductal proliferation. All of the material was formalin fixed and paraffin embedded.

### *Antibody and immunocytochemistry*

Antibody 21N, raised to a synthetic peptide at the c-terminal end of the c-*erbB-2* protein, has been shown to precipitate a 190,000 mol. wt glycoprotein from human cells (Gullick *et al.*, 1987). In a previous study it had been demonstrated that using antibody 21N membrane staining correlated with c-*erbB-2* gene amplification (Gusterson *et al.*, 1987). It has been shown by Dr N. Hynes that membrane staining with this antibody also correlates with increased c-*erbB-2* protein expression (personal communication). On the basis of these

Correspondence: B.A. Gusterson.
Received 1 March 1988; and in revised form, 8 July 1988.

data all of our analyses have been carried out using the antibody titrated against a tumour sample with a 12-fold gene copy number and strong membrane staining. In this study we are therefore aiming to identify the stages in tumour development at which overexpression occurs, the clinical significance of overexpression, and whether overexpression correlates with any identifiable morphological change.

Immunocytochemical staining was carried out using the indirect immunoperoxidase technique. Dewaxed sections were washed in PBS, and endogenous peroxidase blocked with 0.1% phenylhydrosine hydrochloride in PBS for 5 min at room temperature. After washing in PBS, sections were exposed to the first antibody diluted to $3.3\,\mu g\,ml^{-1}$ in PBS for 1.5 h at room temperature. This concentration was based on a previous titration of the antibody against a tumour with a known 12-fold amplification of the c-*erbB-2* gene (Gusterson *et al.*, 1987).

After washing in PBS, sections were incubated with $100\,\mu l$ sheep anti-rabbit peroxidase conjugate (Dako, High Wycombe, Bucks), at a dilution of 1 in 100, for 1 h. The colour was developed using diaminobenzidine (DAB) [10 mg DAB in 100 ml Tris buffer (pH 7.2) 0.1 M, 100 ml M.O, $66\,\mu l$ $H_2O_2$] for 5 min. Sections were dehydrated, cleared and mounted in histomount (National Diagnostics). Control sections included omission of the first antibody and prior absorption of the antibody with peptide ($1\,mg\,ml^{-1}$) at room temperature for 2 min.

### Results

### *Morphological analyses*

Of the 103 infiltrating carcinomas in this study there were 93 ductal carcinomas, 9 lobular carcinomas and 1 medullary. Strong membrane staining was seen in 14 of the ductal carcinomas together with a weak cytoplasmic blush (Figure 1a,b). In two of the lobular carcinomas there was cytoplasmic staining which was both diffuse and granular in type. The single medullary carcinoma was negative. Fifty-seven cases of infiltrating carcinoma had lymph node metastases. The primary tumours were stained in parallel with representative sections of the lymph nodes. Nine primary tumours of the original 14 positive cases had lymph node involvement

**Figure 1** (a) shows strong membrane staining of an infiltrating ductal carcinoma. The apical membranes of the cells adjacent to the lumena are negative. (b) is a parallel section in which the antibody has been prior absorbed with the peptide – this extinguishes all staining (×250).

and an identical pattern of staining was seen in the metastatic deposits. There was no evidence of positive staining in any of the metastases where the primary was negative (Table I). This led us to conclude that overexpression of this protein was not related to the metastatic process. Of the 14 positive cases, five had an *in situ* component and in all instances the staining pattern was identical with the infiltrating component, suggesting that the protein was overexpressed in a small percentage of tumours as a relatively early event in tumorigenesis. In all of the cases where there was strong membrane positivity the tumours were relatively homogeneously positive.

On the basis of the findings in these five intraduct carcinomas it was decided to extend the study to benign lesions and intraduct carcinoma biopsies from a breast screening centre where large numbers of small tumours and atypical lesions were available. The aim was to identify the earliest morphological changes associated with membrane positivity.

Ten of the 24 intraduct carcinomas from the screening centre were positively stained on the membrane. Where a single row of tumour cells lined the ducts the staining was concentrated on the lateral and basal membranes with absence of staining on the luminal surface (Figure 2). Where there was multilayering tumour cells were positive on their adjacent membranes. There was a clear delineation between the positive tumour cells and the adjacent normal cells. In no instances were cells strongly positive on the membranes that were not cytologically malignant. In some micropapillary intraduct carcinomas there was a loss of staining on the cells towards the lumen. This was associated with central necrosis and was interpreted as due to cellular degeneration.

**Table I** 21N staining in primary tumours and lymph node metastases.

| | | *Lymph node* | |
|---|---|---|---|
| | | *+ve* | *−ve* |
| Primary tumour | +ve | 9 | 0 |
| | −ve | 0 | 45 |

**Figure 2** Sections show an intraduct carcinoma. There is strong staining of the pleomorphic tumour cells. Both the normal ductal cells and the surrounding stromal cells are negative. Note the absence of staining of the luminal membrane of the tumour cells (×250).

The results of the benign lesions examined is shown in Table II. Only one case of apocrine metaplasia showed membrane staining and this was weak and on three cells in one cyst wall. The staining was interpreted as due to an artefact produced by fixation. The moderate cytoplasmic staining, which was a consistent finding in apocrine metaplasia in all of the material, when concentrated in a submembrane location as a result of fixation would give the impression of membrane staining. In one case of atypical epitheliosis there was marked cytoplasmic staining of cytologically abnormal cells which were present in a lobule, the significance of which is difficult to assess. There was no evidence of membrane positivity in any of the other cases examined including 22 stellate scars.

### *Clinical correlation*

Data were available on 102 of the carcinoma cases for analysis using Chi-squared test with Yates correction. There was no correlation between the 21N staining and nodal status ($\chi^2=0.2$) or disease free interval ($\chi^2=0.4$) (Table III). When the data were analysed comparing 21N positive and 21N negative patients in relation to disease free survival and overall survival (Figures 3 and 4) overexpression of c-*erbB-2* had no prognostic significance ($\chi^2=1.45$ and 3.37 respectively).

### Discussion

This communication describes the distribution of the c-*erbB-2* protein in the human breast. From these data we have concluded that overexpression of the c-*erbB-2* protein can be visualized immunocytochemically as membrane staining in approximately 14% of breast carcinomas. In this small series there is no significant correlation of staining with tumour progression or lymph node involvement. There was also no relationship to either tumour grade or stage. Although not statistically significant, both the overall survival data and the disease free survival show a trend indicating that 21N positivity may be a poor prognostic factor. A much larger series with a longer follow-up is required to confirm this observation. The morphological studies would suggest that overexpression is an early event in tumorigenesis but in the classical two-stage carcinogenesis model would be considered as post initiation.

In this study overexpression of the c-*erbB-2* protein has an apparently higher incidence (40%) in pure intraduct carcino-

**Table II** 21N staining in intraduct carcinoma and benign breast disease.

| | *Positive* | *Weak cytoplasmic* | *Negative* | *Total* |
|---|---|---|---|---|
| Ductal carcinoma *in situ* | 10 (M) | 8 | 6 | 24 |
| Epitheliosis | 0 | 2 | 4 | 6 |
| Atypical epitheliosis | 1 (C) | 3 | 9 | 13 |
| Radial scars | 1 (C) | 1 | 20 | 22 |
| Papillomata | 0 | 0 | 6 | 6 |
| Adenosis | 0 | 2 | 4 | 6 |
| Blunt duct adenosis | 0 | 0 | 14 | 14 |
| Sclerosing adenosis | 0 | 0 | 4 | 4 |
| Apocrine metaplasia | 1 (C-M) | 24 | 7 | 32 |
| Cysts (including duct ectasia and involution) | 0 | 0 | 22 | 22 |
| Fibroadenomatoid hyperplasia | 0 | 1 | 2 | 3 |
| Fibroadenoma | 0 | 5 | 16 | 21 |

M = Membrane; C = Cytoplasmic.

**Table III** Infiltrating ductal carcinoma.

| | | Disease free interval | |
|---|---|---|---|
| | | *<1 year* | *>5 years* |
| 21N staining | +ve | 6 | 8 |
| | −ve | 27 | 62 |

**Figure 3** Disease-free survival.

**Figure 4** Survival from breast cancer.

mas than infiltrating tumours. The high incidence in these *in situ* lesions is of interest as these cases were all identified at screening. The question has been previously raised whether these radiologically identified lesions would have presented later as clinical cancers or perhaps form a sub-group of tumours which would remain *in situ* or even possibly regress. In fact Millis and Thyme (1975) have raised the interesting possibility that these cases may represent a tumour that has a long dormancy with little clinical importance to the patient. It should be noted that with only 14% of cases positive a relatively small number of tumours were available for analysis and a much larger series would be required to provide a definitive answer.

The weak cytoplasmic staining seen in some cases was seen in all sections when the concentration of the antibody was increased (Gusterson, unpublished). There was not, however, an increase in the number of tumours with membrane staining. Under such conditions all of the cells in the section were stained including inflammatory cells, fibroblasts and smooth muscle in vessel walls. The strong membrane staining observed in some of the tumours, which correlates with overexpression, is also seen in cultured breast carcinoma cells where BT474 cells which have a 125-fold increase in mRNA for c-*erbB-2* compared with MCF7 cells (Kraus *et al.*, 1987) show strong membrane staining while MCF7 cells are only weakly cytoplasmically positive (Gusterson, unpublished).

A number of facts indicate that the c-*erbB-2* protein may have an important role in the pathogenesis of human breast cancer. Firstly the structure of the protein and its membrane localisation suggest that it may be a growth factor receptor (Coussens *et al.*, 1985; Bargmann *et al.*, 1986*a*). Secondly the c-*erbB-2* gene is consistently overexpressed and/or amplified in a significant proportion of infiltrating breast carcinomas in a number of series. The recent demonstration by Di Fiore *et al.* (1987) that the transforming ability of this gene is related to the level of expression of the protein further supports the view that the demonstration of an overexpressed protein in a proportion of breast carcinomas may be related to a specific association with the mechanism of transformation rather than an incidental observation. Further support for a growth regulatory role of c-*erbB-2* is the observation that a monoclonal antibody reactive with the extracellular domain exerted an anti-tumour effect on *neu*-transformed NIH3T3 cells (Drebin *et al.*, 1986). In order to investigate the function of this protein further it will be necessary to produce antibodies that recognise the human external domain. Such antibodies may act as agonists or produce blocking of the putative binding site. They may also provide reagents for radio-localisation studies in a small but easily definable group of breast carcinomas. The absence of membrane staining in normal tissues indicates that this latter approach may be worth pursuing.

Although it is suggested that the c-*erbB-2* protein may be a receptor for an unknown growth factor it is worth considering other possibilities. The sevenless gene in Drosophila encodes a putative transmembrane protein that has a similar structure and sequence homology to hormone receptors including the epidermal growth factor receptor (EGFR) (Hafen *et al.*, 1987). The sevenless protein is required for the formation of the R7 photoreceptor in each ommatidium of the compound eye. It has been suggested that the protein controls this differentiation step by providing positional information, either by cell–cell interaction or through locally diffusable factors (Tomlinson *et al.*, 1987). By analogy it could be speculated that the EGFR, which is known to be expressed on both proliferating and terminally differentiated cells (Gusterson *et al.*, 1984) and the c-*erbB-2* protein with its overexpression on adjacent cell membranes may also be involved in cell–cell interactions. A study of c-*erbB-2* expression in the human embryo and in particular in the developing breast may provide further clues to the function of this molecule.

The Institute of Cancer Research is supported by funds from the Cancer Research Campaign and the Medical Research Council. We are grateful to Dr Nancy Hynes for allowing us to quote her unpublished data.

### References

BARGMANN, C.I., HUNG, M.-C. & WEINBERG, R.A. (1986*a*). The *neu* oncogene encodes an epidermal growth factor receptor-related protein. *Nature*, **319**, 226.

BARGMANN, C.I., HUNG, M.-C. & WEINBERG, R.A. (1986*b*). Multiple independent activations of the *neu* oncogene by a point mutation altering the transmembrane domain of p185. *Cell*, **45**, 649.

COUSSENS, L., YANG-FENG, T.L., LIAO, Y.-C. & 9 others (1985). Tyrosine kinase receptor with extensive homology to EGF receptor shares chromosomal local with *neu* oncogene. *Science*, **230**, 1132.

DI FIORE, P.P., PIERCE, J.H., KRAUS, M.H., SEGATTO, O., RICHTER KING, C. & AARONSON, S.A. (1987). erB-2 is a potent oncogene when overexpressed in NIH/3T3 cells. *Science*, **237**, 178.

DREBIN, J.A., STERN, D.F., LINK, V.C., WEINBERG, R.A. & GREENE, M.I. (1984). Monoclonal antibodies identify a cell-surface antigen associated with an activated cellular oncogene. *Nature*, '**312**, 546.

DREBIN, J.A., LINK, V.C., WEINBERG, R.A. & GREENE, M.I. (1986). Inhibition of tumor growth by a monoclonal antibody reactive with an oncogene-encoded tumor antigen. *Proc. Natl Acad. Sci. USA.*, **83**, 9129.

FUKUSHIGE, S.-I., MATSUBARA, K.-I., YOSHIDA, M. & 5 others (1986). Localisation of a novel v-*erb*B-related gene. c-*erb*B-2, on human chromosome 17 and its amplification in a gastric cancer cell line. *Mol. Cell. Biol.*, **5**, 1442.

GULLICK, W.J., BERGER, M.S., BENNETT, P.L.P., ROTHBARD, J.B. & WATERFIELD, M.D. (1987). Expression of the c-erbB-2 protein in normal and transformed cells. *Int. J. Cancer*, **40**, 246.

GUSTERSON, B., COWLEY, G., SMITH, J.A. & OZANNE, B. (1984). Cellular localisation of human epidermal growth factor receptor. *Cell Biol. Int. Rep.*, **8**, 649.

GUSTERSON, B.A., GULLICK, W.J., VENTER, D.J. & 5 others (1987). Immunohistochemical localization of c-*erbB-2* in breast carcinoma. *Mol. Cell. Probes*, **1**, 383.

HAFEN, E., BASLER, K., EDSTROEM, J.-E. & RUBIN, G.M. (1987). *Sevenless*, a cell-specific homeotic gene of Drosophila, encodes a putative transmembrane receptor with a tyrosine kinase domain. *Science*, **236**, 55.

KRAUS, M.H., POPESCU, N.C., AMSBAUGH, S.C. & RICHTER KING, C. (1987). Overexpression of the EGF receptor-related proto-oncogene *erb*B-2 in human mammary tumour cell lines by different molecular mechanisms. *EMBO J.*, **6**, 605.

MILLIS, R.R. & THYME, G.S.J. (1975). *In situ* intraduct carcinoma of the breast: a long term follow-up study. *Br. J. Surg.*, **62**, 957.

PADHY, L.C., SHIH, C., COWING, D., FINKELSTEIN, R. & WEINBERG, R.A. (1982). Identification of a phosphoprotein specifically induced by the transforming DNA of rat neuroblastomas. *Cell*, **28**, 865.

SLAMON, D.J., CLARK, G.M., WONG, S.G., LEVIN, W.J., ULLRICH, A. & McGUIRE, W.L. (1987). Human breast cancer: Correlation of relapse and survival with amplification of the HER-2/*neu* oncogene. *Science*, **235**, 177.

TOMLINSON, A., BOWTELL, D.D.L., HAFEN, E. & RUBIN, G.M. (1987). Localization of the sevenless protein, a putative receptor for positional information in the eye imaginal disc for Drosophila. *Cell*, **51**, 143.

VAN DE VIJVER, M., CAN DE BERSSELAAR, R., DEVILEE, P., CORNELISSE, C., PETERSE, J. & NUSSE, R. (1987). Amplification of the *neu* (c-*erb*B-2) oncogene in human mammary tumors is relatively frequent and is often accompanied by amplification of the linked c-*erb*A oncogene. *Mol. Cell. Biol.*, **7**, 2019.

VARLEY, J.M., SWALLOW, J.E., BRAMMAR, W.J., WHITTAKER, J.L. & WALKER, R. (1987). Alterations to either c-erbB-2 (neu) or c-myc proto-oncogenes in breast carcinomas correlate with poor short-term prognosis. *Oncogene*, **1**, 423.

VENTER, D.J., TUZI, N.L., KUMAR, S. & GULLICK, W.J. (1987). Overexpression of the c-*erbB-2* onco-protein in human breast carcinomas: immunohistological assessment correlates with gene amplication. *Lancet*, **ii**, 69.

ZHOU, D., BATTIFORA, H., YOKOTA, J., YAMAMOTO, T. & CLINE, M.J. (1987). Association of multiple copies of the c-*erbB-2* oncogene with spread of breast cancer. *Cancer Res.*, **47**, 6123.


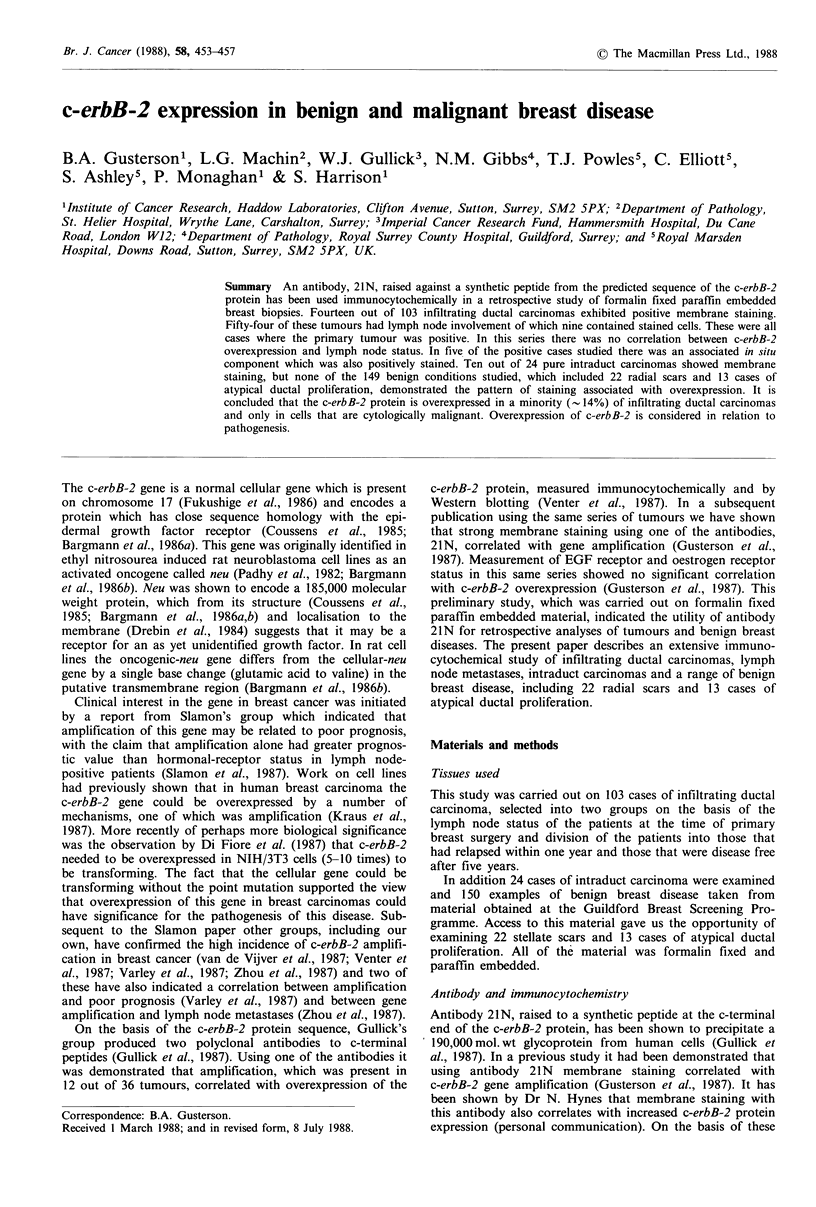

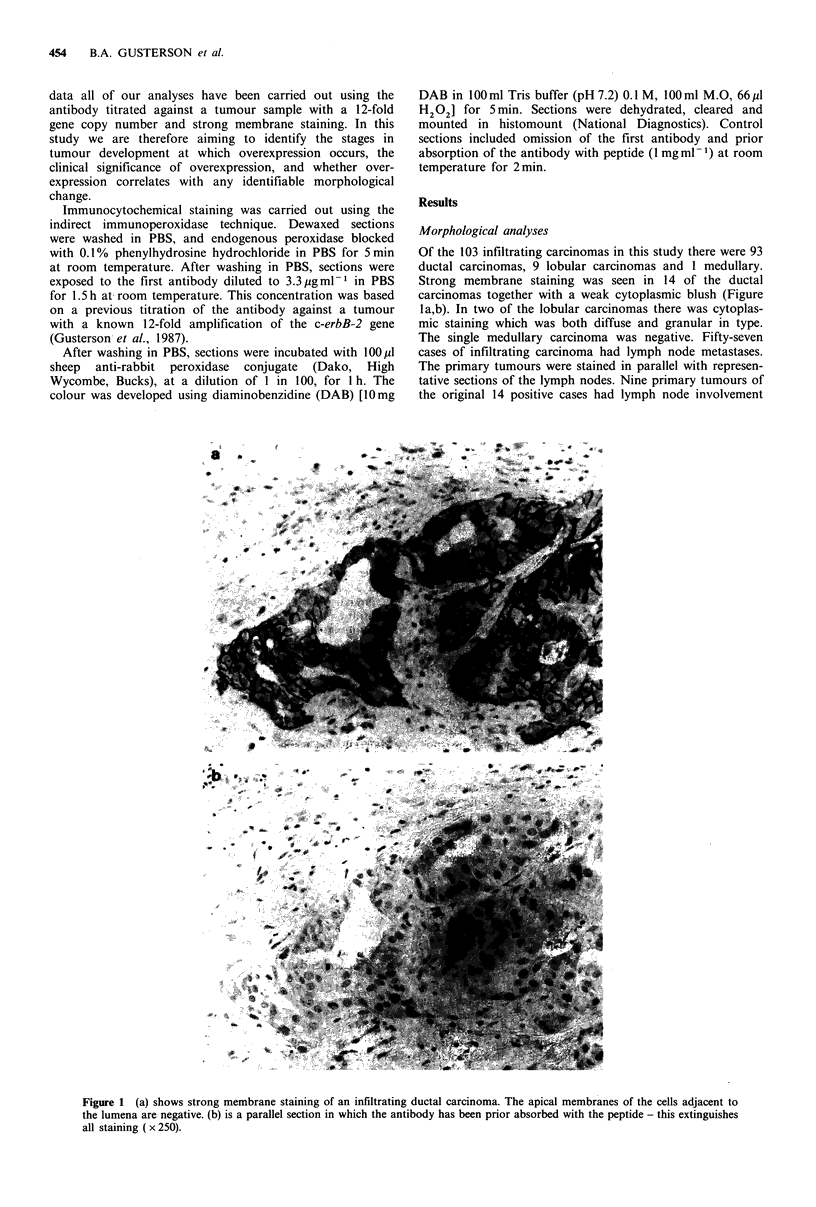

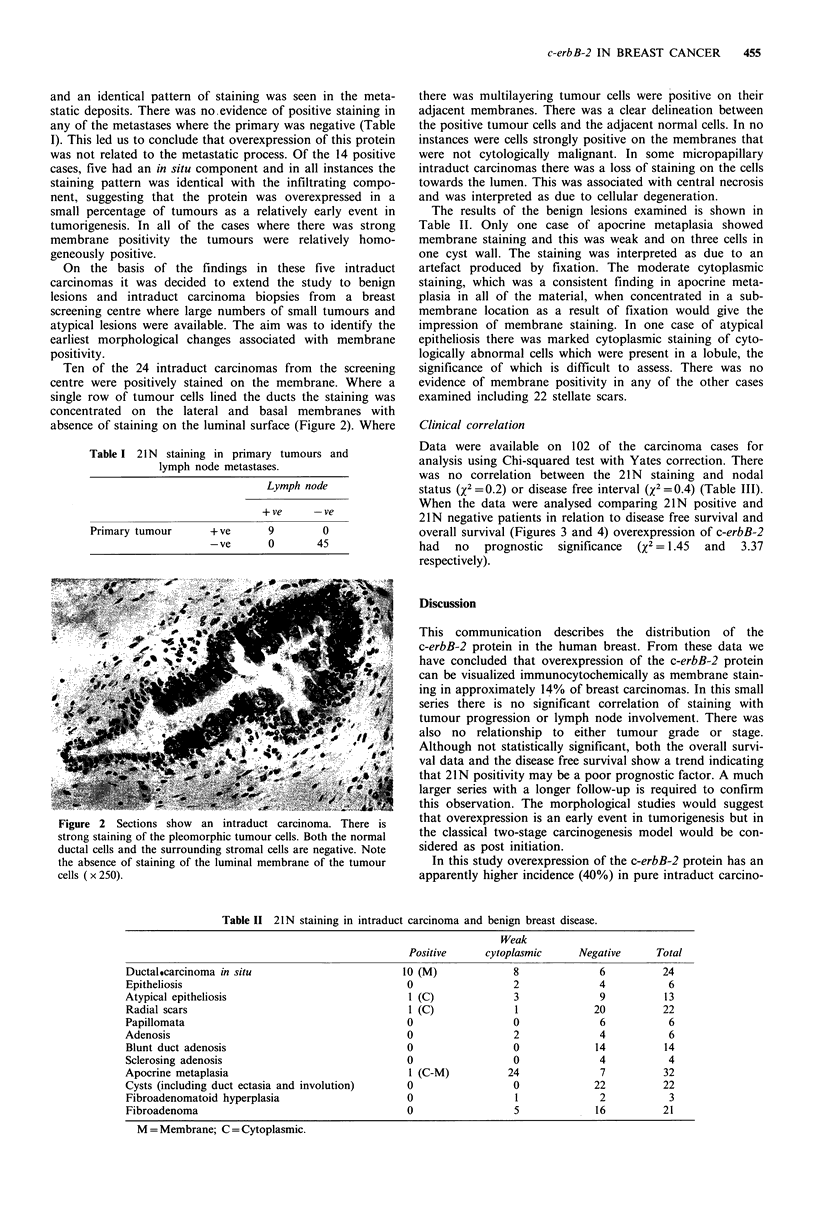

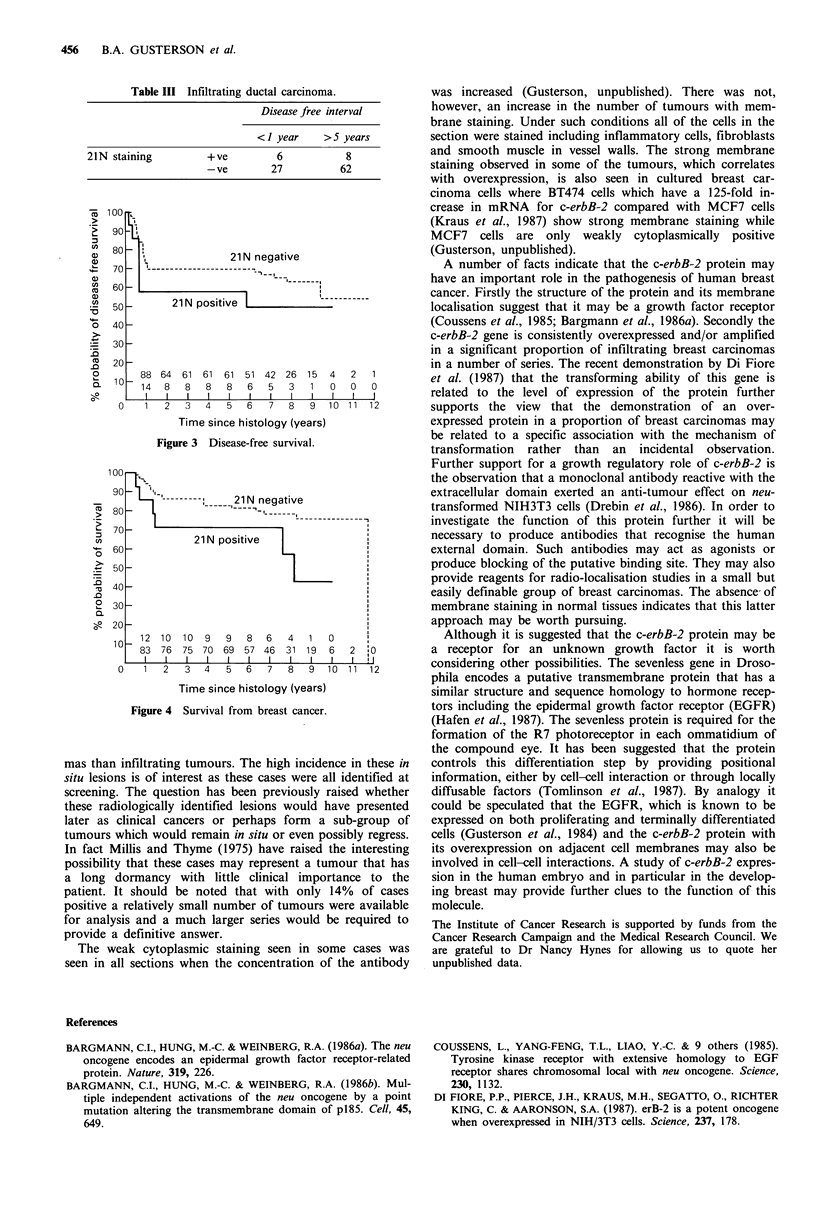

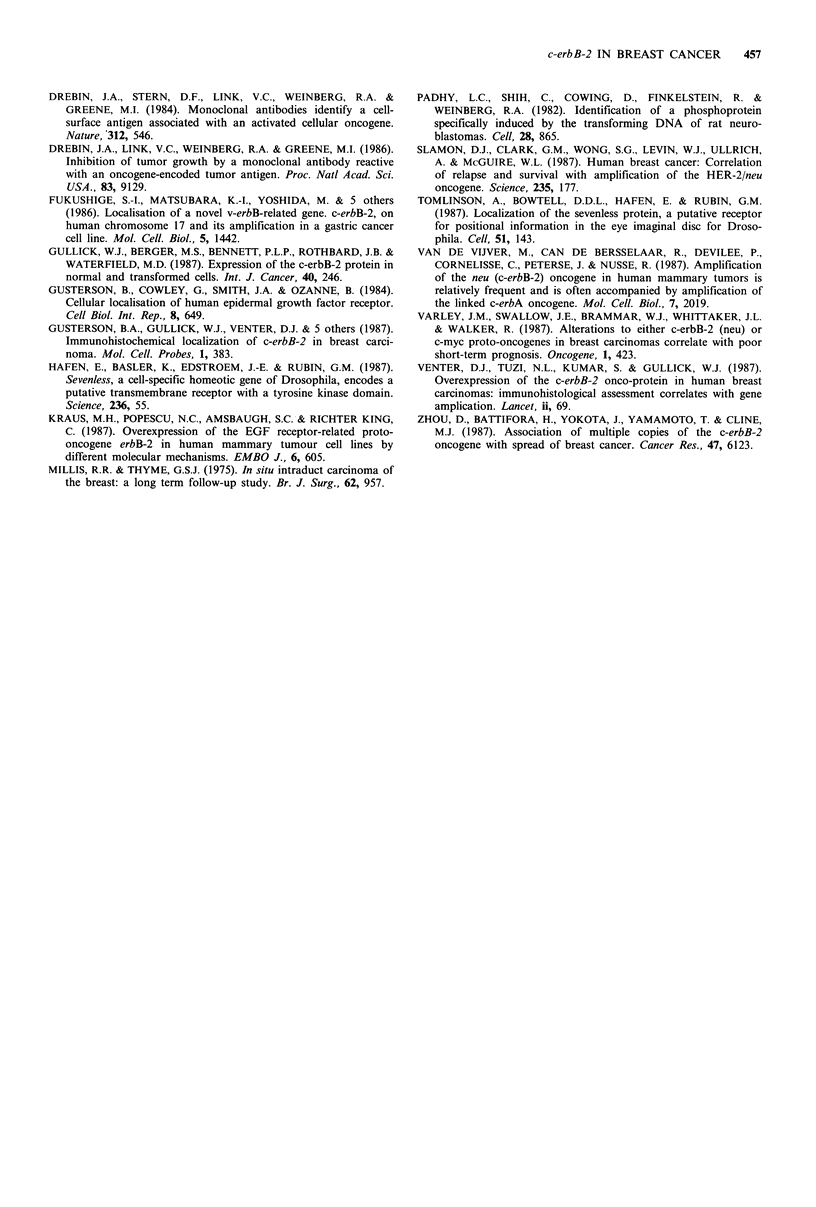


## References

[OCR_00490] Bargmann C. I., Hung M. C., Weinberg R. A. (1986). Multiple independent activations of the neu oncogene by a point mutation altering the transmembrane domain of p185.. Cell.

[OCR_00485] Bargmann C. I., Hung M. C., Weinberg R. A. (1986). The neu oncogene encodes an epidermal growth factor receptor-related protein.. Nature.

[OCR_00496] Coussens L., Yang-Feng T. L., Liao Y. C., Chen E., Gray A., McGrath J., Seeburg P. H., Libermann T. A., Schlessinger J., Francke U. (1985). Tyrosine kinase receptor with extensive homology to EGF receptor shares chromosomal location with neu oncogene.. Science.

[OCR_00502] Di Fiore P. P., Pierce J. H., Kraus M. H., Segatto O., King C. R., Aaronson S. A. (1987). erbB-2 is a potent oncogene when overexpressed in NIH/3T3 cells.. Science.

[OCR_00515] Drebin J. A., Link V. C., Weinberg R. A., Greene M. I. (1986). Inhibition of tumor growth by a monoclonal antibody reactive with an oncogene-encoded tumor antigen.. Proc Natl Acad Sci U S A.

[OCR_00527] Gullick W. J., Berger M. S., Bennett P. L., Rothbard J. B., Waterfield M. D. (1987). Expression of the c-erbB-2 protein in normal and transformed cells.. Int J Cancer.

[OCR_00537] Gusterson B. A., Gullick W. J., Venter D. J., Powles T. J., Elliott C., Ashley S., Tidy A., Harrison S. (1987). Immunohistochemical localization of c-erbB-2 in human breast carcinomas.. Mol Cell Probes.

[OCR_00532] Gusterson B., Cowley G., Smith J. A., Ozanne B. (1984). Cellular localisation of human epidermal growth factor receptor.. Cell Biol Int Rep.

[OCR_00542] Hafen E., Basler K., Edstroem J. E., Rubin G. M. (1987). Sevenless, a cell-specific homeotic gene of Drosophila, encodes a putative transmembrane receptor with a tyrosine kinase domain.. Science.

[OCR_00548] Kraus M. H., Popescu N. C., Amsbaugh S. C., King C. R. (1987). Overexpression of the EGF receptor-related proto-oncogene erbB-2 in human mammary tumor cell lines by different molecular mechanisms.. EMBO J.

[OCR_00554] Millis R. R., Thynne G. S. (1975). In situ intraduct carcinoma of the breast: a long term follow-up study.. Br J Surg.

[OCR_00558] Padhy L. C., Shih C., Cowing D., Finkelstein R., Weinberg R. A. (1982). Identification of a phosphoprotein specifically induced by the transforming DNA of rat neuroblastomas.. Cell.

[OCR_00564] Slamon D. J., Clark G. M., Wong S. G., Levin W. J., Ullrich A., McGuire W. L. (1987). Human breast cancer: correlation of relapse and survival with amplification of the HER-2/neu oncogene.. Science.

[OCR_00570] Tomlinson A., Bowtell D. D., Hafen E., Rubin G. M. (1987). Localization of the sevenless protein, a putative receptor for positional information, in the eye imaginal disc of Drosophila.. Cell.

[OCR_00583] Varley J. M., Swallow J. E., Brammar W. J., Whittaker J. L., Walker R. A. (1987). Alterations to either c-erbB-2(neu) or c-myc proto-oncogenes in breast carcinomas correlate with poor short-term prognosis.. Oncogene.

[OCR_00589] Venter D. J., Tuzi N. L., Kumar S., Gullick W. J. (1987). Overexpression of the c-erbB-2 oncoprotein in human breast carcinomas: immunohistological assessment correlates with gene amplification.. Lancet.

[OCR_00595] Zhou D., Battifora H., Yokota J., Yamamoto T., Cline M. J. (1987). Association of multiple copies of the c-erbB-2 oncogene with spread of breast cancer.. Cancer Res.

[OCR_00578] van de Vijver M., van de Bersselaar R., Devilee P., Cornelisse C., Peterse J., Nusse R. (1987). Amplification of the neu (c-erbB-2) oncogene in human mammmary tumors is relatively frequent and is often accompanied by amplification of the linked c-erbA oncogene.. Mol Cell Biol.

